# The Inevitable Fate of *Tetranychus urticae* on Tomato Plants Treated with Entomopathogenic Fungi and Spinosad

**DOI:** 10.3390/jof11020138

**Published:** 2025-02-12

**Authors:** Waqas Wakil, Maria C. Boukouvala, Nickolas G. Kavallieratos, Aqsa Naeem, Dionysios Ntinokas, Muhammad Usman Ghazanfar, Pasco B. Avery

**Affiliations:** 1 Department of Entomology, University of Agriculture, Faisalabad 38040, Pakistan; aqsanaeem231@gmail.com; 2 Senckenberg German Entomological Institute, D-15374 Müncheberg, Germany; 3 Laboratory of Agricultural Zoology and Entomology, Department of Crop Science, Agricultural University of Athens, 75 Iera Odos Str., 11855 Athens, Greece; mbouk@aua.gr (M.C.B.); dntinokas@aua.gr (D.N.); 4 Department of Plant Pathology, College of Agriculture, Sargodha University, Sargodha 40100, Pakistan; usmanghazanfar@uos.edu.pk; 5 Indian River Research and Education Center, Department of Entomology and Nematology, Institute for Agricultural Sciences, University of Florida, Ft. Pierce, FL 34945, USA; pbavery@ufl.edu

**Keywords:** two-spotted spider mite, combination treatment applications, *Beauveria bassiana*, *Metarhizium robertsii*, spynosins

## Abstract

*Tetranychus urticae* (Acari: Tetranychidae) is a pervasive and damaging mite pest of tomato crops, leading to important economic losses globally. This study evaluated the acaricidal efficacy of spinosad, alone and in combination with *Beauveria bassiana* (*Bb*) WG-21 and *Metarhizium robertsii* (*Mr*) WG-04, in the laboratory (application to tomato leaf discs) and greenhouse (application to tomato plants), considering mortality and establishment, respectively. The combination treatments of *Bb* WG-21 or *Mr* WG-04 with spinosad achieved 100% mortality of *T. urticae* nymphs within 2 days on leaf discs, while individual applications of each control agent resulted in lower mortalities, ranging between 62.91 and 86.25% after 3 days. The paired treatment of *Mr* WG-04 + spinosad killed all exposed adults within 5 d, while that of *Bb* WG-21 + spinosad achieved the same results after 7 d. However, spinosad, *Mr* WG-04, and *Bb* WG-21 alone killed ≥77.08% of adults after 7 d. In the greenhouse, the combination treatment of WG-04 + spinosad deterred the presence of *T. urticae* (adults, immatures, and eggs) on either surface of the tomato leaves, while *Bb* WG-21 + spinosad suppressed the populations only on the adaxial surface. These findings indicate that combined treatments of the tested EPF + spinosad, especially *Mr* WG-04, on tomato plants under greenhouse conditions can provide substantially enhanced control of *T. urticae* life stages compared to each treatment applied alone.

## 1. Introduction

The tetranychid *Tetranychus urticae* Koch (Acari) is widely recognized as a highly adaptable generalist herbivore [1], ranking among the most destructive pests affecting field and greenhouse crops [2,3,4,5]. This pest has an extensive host range that includes >1400 plant species from >200 plant families and is found in various geographical regions, such as Australasian, Oriental, Afrotropical, Neotropical, Palaearctic, and Nearctic [1,5,6,7]. *Tetranychus urticae* also stands out as a highly destructive pest with a significant economic impact on tomato crops worldwide [2,8,9]. Concretely, 10–50% of tomato production losses worldwide are due to *T. urticae* [10]. It feeds on plant sap by puncturing leaves with its mouthparts, causing functional damage to foliage and increasing its vulnerability to viruses and pathogens [4,11]. In addition, due to its short life cycle, *T. urticae* can quickly inflict severe damage on plants resulting in leaf streaking [12,13,14]. In cases of heavy infestation, this damage can lead to defoliation, stunted plant growth, and complete plant deterioration, and even the death of plants, severely impacting both the quality and yield of affected crops [14,15].

Chemical acaricides have traditionally been the main approach for preventing *T. urticae* from inflicting substantial economic damage [16,17]. However, due to their repeated and intensive use, *T. urticae* has developed resistance to nearly all classes of acaricides [18,19,20,21]. *Tetranychus urticae* resistance to chemical acaricides also has developed due to its arrhenotokous parthenogenesis reproduction, which increases genetic diversity and promotes the survival of resistant individuals [18]. In addition, the short life span of *T. urticae*, high offspring production rate, and multiple annual generations facilitate the rapid spread of resistance genes in its populations [18,22].

Due to the ineffectiveness of chemical control methods and environmental impacts, research efforts are increasingly focused on alternative strategies against *T. urticae*, including the use of entomopathogenic fungi (EPF) [10,23,24,25,26]. EPF, such as *Metarhizium robertsii* J.F. Bisch., Rehner & Humber (Hypocreales: Clavicipitaceae), and *Beauveria bassiana* (Balsamo-Crivelli) Vuillemin (Hypocreales: Cordycipitaceae) can infect and kill *T. urticae* without affecting the non-target organisms [10,25,26,27,28]. EPF are also safe for humans and do not cause any harm to the environment [10,29]. EPF infect and kill insects through a multi-step infection process [30]. Initially, fungal spores (conidia) come into contact with the cuticle of the host and attach, germinate, and produce enzymes to break down the host cuticle [31,32,33,34]. Once inside the host, the fungal hyphae proliferate and enter the hemocoel, where they produce blastospores or hyphal bodies that spread throughout the host’s internal body, releasing toxins [35,36]. The fungus emerges from the cadaver and sporulates, releasing new conidia to infect other hosts [35,37].

Spinosad is a bioinsecticide, derived from the natural fermentation of *Saccharopolyspora spinosa* Mertz & Yao (Pseudonocardiales: Pseudonocardiaceae), a soil-borne bacterium, consisting of a mixture of two macrocyclic lactones, spynosins A and D [38,39]. Spinosad operates by selectively targeting the nervous system’s nicotinic acetylcholine receptors (nAChRs) in pests, functioning as an allosteric modulator. This action rapidly stimulates both the γ-aminobutyric acid (GABA) receptors and nAChRs, causing intense nervous excitation that culminates in paralysis and, eventually, death of the organism [39,40,41]. Spinosad has widespread use against economically important insects from various orders (i.e., Lepidoptera, Coleoptera, Thysanoptera, and Diptera) across several agricultural crops, stored products, and ornamental plants [42,43,44]. For instance, spinosad has been successfully applied to control larval stages of pest species within Coleoptera and Lepidoptera in stored products, adult Diptera in certain crops, and even mosquito larvae in disease vector management efforts [39,45,46,47]. This active ingredient is not intended for the management of spider mites. However, their inadvertent exposure to spinosad does occur, particularly in greenhouse environments due to the widespread use of this pesticide to control caterpillars, leafminers, and thrips [48,49,50,51,52].

It is widely known that the combined use of EPF with pesticides may enhance the effectiveness of pest control (insects and mites) and support integrated pest management (IPM) strategies [26,53,54,55,56]. Furthermore, this combined treatment approach helps mitigate the development of pest resistance and promotes environmentally friendly pest management by reducing the amount of pest exposure to broad-spectrum single-mode-of-action synthetic chemicals [26,53,54,56,57,58]. A thorough investigation of the published literature revealed that the combination of *M. robertsii* or *B. bassiana* with spinosad against *T. urticae* has not been studied in comparison to the single treatments applied alone (i.e., EPF or chemical). Therefore, the aim of this study was to investigate the effectiveness of the combined application of spinosad with *M. robertsii* or *B. bassiana* vs. each treatment alone against different life stages (adults and immatures) of *T. urticae* under laboratory and greenhouse conditions.

## 2. Materials and Methods

### 2.1. Tetranychus urticae

*Tetranychus urticae* individuals came from a laboratory colony nurtured at the University of Agriculture, Faisalabad (UAF). The colony originated from mites collected from a natural population that was homogenized [59] and maintained for the past 4 years on tomato plants (*Solanum lycopersicum* L. cv. Moneymaker) inside MIR-254-PE incubators (Panasonic, Kadoma, Japan) set at 26 °C with 65% relative humidity (RH) using daylight lamps under a 16:8 h light (L)/dark (D) photoperiod [60]. Previous studies have reported notable differences in the demography of *T. urticae* feeding on the sap of whole leaves compared to leaf discs [61]. Thus, to minimize this variation and ensure an accurate assessment of *T. urticae* life stages, the rearing method described by Puspitarini et al. [62] was applied for all experiments.

### 2.2. Isolates of Metarhizium robertsii and Beauveria bassiana

The isolates of the EPF *Mr* WG-04 and *Bb* WG-21 corresponding to *M. robertsii* and *B. bassiana*, respectively, were selected for the experiments. *Mr* WG-04 and *Bb* WG-21 were recovered from soil samples collected from Chichawatni, Punjab [63] and Lahore, Punjab [64], respectively. Both EPF isolates were maintained at UAF’s microbial culture collection inside Petri dishes that had potato dextrose agar-PDA (Sigma-Aldrich, Taufkirchen, Germany), stored at 4 °C [25,26]. Fungal isolates were cultured separately on Petri dishes (Ø 6 cm × 1.5 cm) that contained Sabouraud dextrose agar (BD-Difco, Franklin Lakes, NJ, USA) + 1% yeast (SDAY). The cultured dishes were closed, sealed with Nescofilm^®^ (Azwell, Osaka, Japan), and incubated in a MIR-254-PE incubator in complete darkness for 14 days at 24 °C [65]. Two weeks after inoculation, these dishes produced a substantial quantity of conidia. A sterilized glass Drigalski spatula (LaborXing, Shenzhen, China) was used to harvest the dry conidia from the SDAY surface, transferring them into a sterile tube (10 mL). Some of the collected conidia were then suspended inside a Falcon tube (50 mL) containing 30 mL of 0.05% *v*/*v* of sterile polysorbate (Tween 80) solution (Merck & Co., Inc., Rahway, NJ, USA) to minimize clumping, as noted by Ortucu et al. [66]. The conidial suspension was vortexed for 5 min using a vortexer (Velp Scientifica srl, Usmate, Italy) with eight sterile glass beads. The slurry was passed through a sterilized cheesecloth (double layered) to remove any mycelial clumps into a Falcon tube (50 mL volume) [67,68]. The appropriate concentration was determined using a Neubauer hemocytometer (Marienfeld, Germany) with a microscope at 400× magnification [69].

The germination of the conidia was evaluated in two 6 cm diameter dishes containing SDAY by inoculating 0.1 mL of the conidia suspension (i.e., 1 × 10^6^ conidia/mL). The dishes were sealed with Nescofilm (Azwell, Osaka, Japan) and put in incubator for 18 h set at 25 °C, under a 14:10 h L/D photoperiod. After incubation, each dish was unsealed, the top cover removed, and sterile coverslips were placed on top of the agar inside the dishes. For each fungal isolate, two hundred conidia per EPF isolate and dish were counted [25,26]. If the length of conidia germ tube was twice the diameter, their germination was counted as viable when viewed at 400× magnification using a light microscope (Euromex BB.1152-PLi, Euromex Microscopen bv, Arnhem, The Netherlands) as described by the method of Wakil et al. [25,26]. Prior to the experiments, the conidia viability for all isolates was >92%.

### 2.3. Insecticide

The spinosad formulation Tracer 240 SC, provided by Dow Agro Sciences (Karachi, Pakistan) containing 240 g/L of the active ingredient (a.i.), was used in the tests. The insecticide was dissolved in Tween 80 (0.05%) solution.

### 2.4. Effects of Entomopathogenic Fungal Isolates and Spinosad Alone and in Combination Against T. urticae Under Laboratory Conditions

The efficacy of *M*. *robertsii* (*Mr* WG-04), *B*. *bassiana* (*Bb* WG-21) and spinosad alone and in combination was evaluated against *T*. *urticae* individuals (nymphs, adult females) under laboratory conditions. The experiments were conducted in plastic Petri dishes (Ø 50 mm × 15 mm) (3 subreplicates/test), holding a tomato (var. “Moneymaker”) leaf disc (20 mm diameter) according to the design of Marcossi et al. [70]. This variety was selected for its strong potential and widespread cultivation by Pakistani tomato growers [25,26,71,72]. Prior to spraying, a cork borer was used to prepare the leaf discs. Inside each dish, a damp tissue paper (Ø 3 cm) was placed, then, a leaf disc was placed on top of the tissue paper with the abaxial side facing up and surrounded by a wet cotton swab to maintain moisture [25,26,73]. Groups of twenty *T. urticae* adult females (1 day old) or nymphs (1 day old) [26] were collected and individually placed on each tomato leaf disc [74]. We selected adult females since they are the main vehicle of reproduction. A Master Multipurpose Airbrush (Las Vegas, NM, USA) was then used to spray the leaf discs containing the mites with 1 mL of *Mr* WG-04 or *Bb* WG-21 isolate suspension containing 1 × 10^8^ conidia and allowed to air-dry for 3 h. After preliminary experimentation, examining a range of fungal concentrations from 1 × 10^4^ to 1 × 10^9^, we selected the fungal dose rate that gave approximately 35–55% mortality (nymphs and adult females) on the first day. In the case of spinosad, leaf discs were dipped in a solution at 0.15 mg spinosad/L for 15 sec and allowed to air-dry for 3 h [75]. We mixed spinosad with Tween 80 (0.05%) solution to receive our appropriate concentration. Then, the dry discs were placed in the dishes as described above and groups of 20 adult females or nymphs were released in each disc. Concerning the combination treatment of *Bb* WG-21 or *Mr* WG-04 with spinosad, each leaf disc was first treated with the insecticide alone (leaf dip technique), as described above. The treated dry discs were then sprayed with each fungal isolate at 1 × 10^8^ conidia/mL as described above. Control leaf discs were treated with 1 mL of a Tween 80 (0.05%) solution [26,66,73] with a different airbrush of the same model reserved for treating controls and allowed to air-dry 3 h. Nescofilm was used to seal the dishes, which were then placed in an incubator set at 25 °C with 60% RH, under a 16:8 h L/D photoperiod [26,76,77]. Mortality data were obtained after observing nymphs and adults 1, 2, 3, and 3, 5, 7 days post treatment, respectively, according to Wakil et al. [26]. Per exposure, separate tomato leaf discs containing either nymphs or adult females of *T. urticae* were prepared. Dead individuals were assessed using a Leica stereomicroscope (Wild M3B, Heerbrugg, Switzerland). Adults or nymphs were considered dead if they showed no movement in their appendages after gently prodding with a camel hairbrush [73]. The entire experimental procedure described above was performed 4 times in a completely randomized design, where new dishes, tomato leaf discs, insecticidal solutions, EPF suspensions, and mite individuals were prepared for each test (i.e., 4 replications × 3 sub-replicates × 6 (treatments + control) × 2 life stages).

### 2.5. Effects of Entomopathogenic Fungal Isolates and Insecticide Against T. urticae Under Greenhouse Conditions

**Figure 1:**
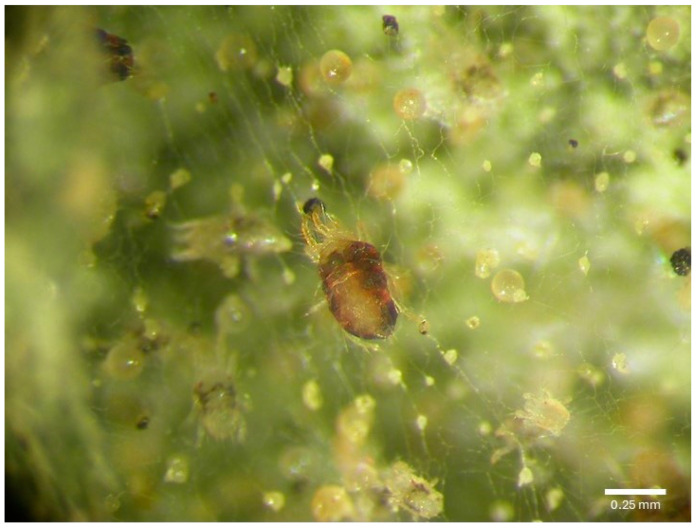
Adult female of *Tetranychus urticae*.

Seeds of the tomato variety “Moneymaker” were sown in seedling trays at a rate of two seeds/well and watered daily. Twenty-one days post sowing, each seedling was transplanted individually into 3 L plastic pots (one plant per pot) with sphagnum peat moss. The pots were then transferred to chambers at 25 °C, with 65% RH, under a 16:8 h L/D photoperiod [26,78]. The plants were irrigated every three days. After 15 days, two fertilizers were applied, as recommended by Wakil et al. [26]. After 20 days, plants showing uniform growth traits were selected for the bioassays [26,60]. For each treatment and control, three pots containing three plants in each pot (3 subreplications) were chosen. Each plant had ten to fifteen leaves [26]. A solution (15 mL) of spinosad (30 mg/L H_2_O) or a 15 mL conidial suspension (1 × 10^8^ conidia/mL) of either *Mr* WG-04 or *Bb* WG-21 was sprayed until runoff on the plants in their respective groups using a handheld sprayer (Kissan Ghar, Sargodha, Pakistan). Each plant was sprayed thoroughly with each EPF suspension or spinosad solution until runoff to ensure uniform coverage [73]. Control plants were treated until runoff with 15 mL of a 0.05% Tween 80 solution [66,73]. For the combination treatments, *Mr* WG-04 or *Bb* WG-21 with spinosad, two consecutive sprays were applied [26,79]. The plants were initially sprayed with 15 mL of the spinosad solution (30 mg/L H_2_O) until runoff and allowed to air-dry for 24 h. This was followed by a second spray with 15 mL of each EPF suspension (1 × 10^8^ conidia/mL) until runoff [26,79]. All plants were allowed to air-dry for 24 h after being sprayed. Using a fine hairbrush, 20 adult females of *T. urticae* were gently placed on the three apical leaflets of the 2nd oldest true leaf on each plant [26]. After 21 days from the initial infestation, all leaves on each plant were cut and examined with a Leica stereomicroscope to assess mite establishment, recording adults (Figure 1), immatures, and eggs of *T. urticae* on both leaf surfaces (adaxial and abaxial) [26,80]. The experiment followed a randomized complete block design (RCBD), which was repeated 4 times, each trial involving new potted plants, mites, insecticidal solutions, and fungal suspension (i.e., 4 replications × 3 subreplicates × 6 (treatments + control) × 2 life stages).

### 2.6. Statistical Analysis

Prior to analysis, the data set was log (x + 1)-transformed to normalize the variance [81,82]. Abbott’s formula was used to correct mortality data [83]. For laboratory assays, the main effects were life stage, treatment, and interval with mortality being the response variable. For data on the number of mites (i.e., eggs, immatures, and adults) per leaf in the greenhouse experiments, the main effects were treatment, life stage, and leaf surface position (abaxial or adaxial). Mite presence on the adaxial or abaxial surface of leaves consisted of the response variable. In all cases, data analysis was performed by a three-way ANOVA, accounting for the main effects and their interactions. Control mortality was below 5%. Mean separation was carried out using the Tukey HSD test at a 5% significance level [84]. The “Minitab” statistical package was used for all statistical analyses [85].

## 3. Results

### 3.1. Subsection Mortality of Tetranychus urticae Life Stages in the Laboratory Tests

**Figure 2:**
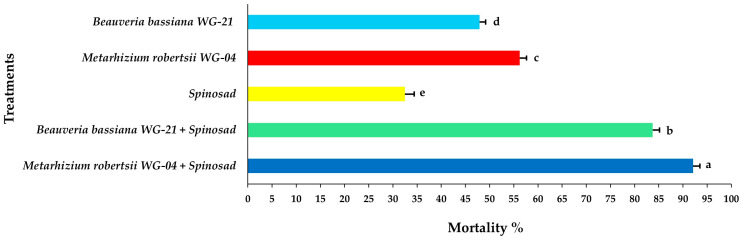
Mean mortality% ± Standard Error (SE) of *Tetranychus urticae* nymphs 1 day after exposure to tomato leaf discs treated with EPF *Beauveria bassiana* WG-21, *Metarhizium robertsii* WG-04 or spinosad, and in combinations (*Beauveria bassiana* WG-21 + Spinosad and *Metarhizium robertsii* WG-04 + Spinosad). Different letters indicate significant differences.

**Figure 3:**
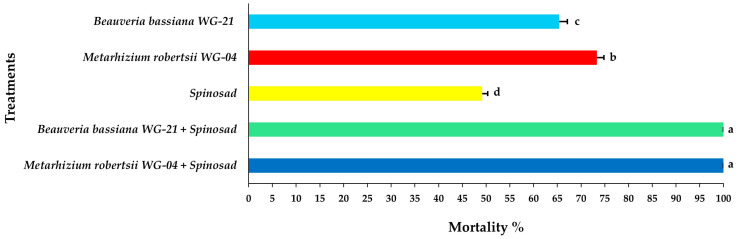
Mean mortality% ± Standard Error (SE) of *Tetranychus urticae* nymphs 2 days after exposure to tomato leaf discs treated with EPF *Beauveria bassiana* WG-21, *Metarhizium robertsii* WG-04 or spinosad, and in combinations (*Beauveria bassiana* WG-21 + Spinosad and *Metarhizium robertsii* WG-04 + Spinosad). Different letters indicate significant differences.

**Figure 4:**
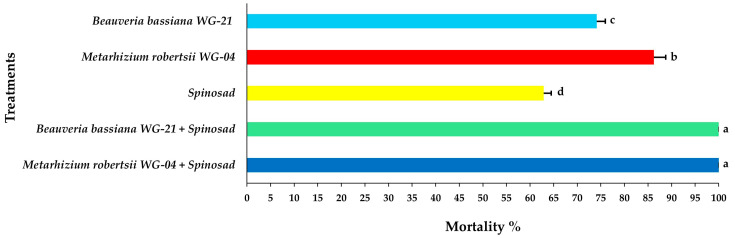
Mean mortality% ± Standard Error (SE) of *Tetranychus urticae* nymphs 3 days after exposure to tomato leaf discs treated with EPF *Beauveria bassiana* WG-21, *Metarhizium robertsii* WG-04 or spinosad, and in combinations (*Beauveria bassiana* WG-21 + Spinosad and *Metarhizium robertsii* WG-04 + Spinosad). Different letters indicate significant differences.

From the ANOVA results, all of the effects alone (interval, life stage, treatment) and in various combinations of interactions were significant; however, only the associated interaction “interval × stage” combination was not significant (Table 1). On the first day of observation, the nymphal mortality ranged from 32.50 to 56.25% after individual applications of either EPF isolates or spinosad alone applied on the tomato leaf discs (Figure 2, Table S1). In contrast, the combined applications of EPF with spinosad resulted in 83.75 and 92.08%, for *Bb* WG-21 + spinosad and *Mr* WG-04 + spinosad, respectively, at 1 day post treatment. Two days after treatment (DAT), complete mortality (100%) was recorded in both fungal–spinosad combinations, whereas mortality ranged between 49.16 and 73.33% for spinosad and fungal treatments alone (Figure 3, Table S1). Three DAT, *Mr* WG-04 (86.25%) killed significantly more immatures than *Bb* WG-21 (74.16%) and spinosad (62.91%) (Figure 4, Table S1).

**Figure 5:**
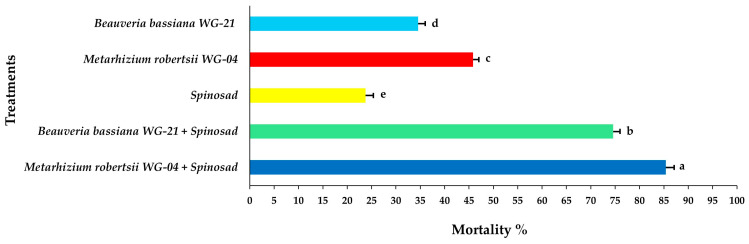
Mean mortality% ± Standard Error (SE) of *Tetranychus urticae* adults 3 days after exposure to tomato leaf discs treated with EPF *Beauveria bassiana* WG-21, *Metarhizium robertsii* WG-04 or spinosad, and in combinations (*Beauveria bassiana* WG-21 + Spinosad and *Metarhizium robertsii* WG-04 + Spinosad). Different letters indicate significant differences.

**Figure 6:**
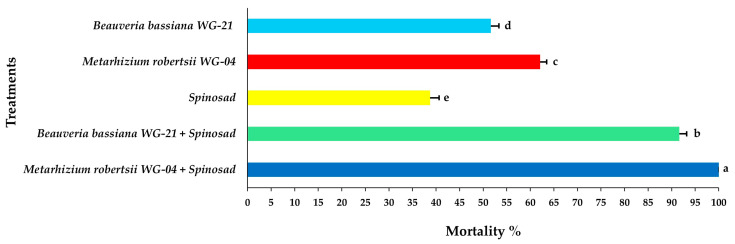
Mean mortality% ± Standard Error (SE) of *Tetranychus urticae* adults 5 days after exposure to tomato leaf discs treated with EPF *Beauveria bassiana* WG-21, *Metarhizium robertsii* WG-04 or spinosad, and in combinations (*Beauveria bassiana* WG-21 + Spinosad and *Metarhizium robertsii* WG-04 + Spinosad). Different letters indicate significant differences.

**Figure 7:**
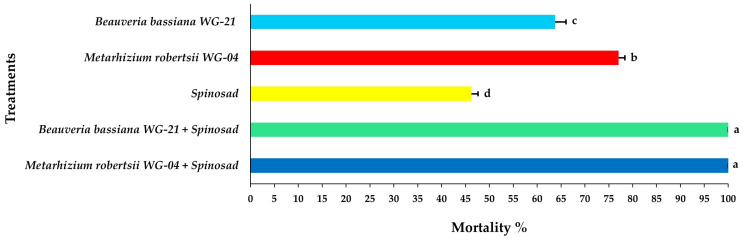
Mean mortality% ± Standard Error (SE) of *Tetranychus urticae* adults 7 days after exposure to tomato leaf discs treated with EPF *Beauveria bassiana* WG-21, *Metarhizium robertsii* WG-04 or spinosad, and in combinations (*Beauveria bassiana* WG-21 + Spinosad and *Metarhizium robertsii* WG-04 + Spinosad). Different letters indicate significant differences.

Concerning the mortality of adult females, significant differences were noted among treatments at 3 DAT, with the combined application of *Mr* WG-04 + spinosad showing the highest mortality (85.41%) and the single application of spinosad the lowest (23.75%) (Figure 5, Table S1). After 5 DAT, the combination of *Mr* WG-04 + spinosad killed all (100%) exposed females on the leaf disc. In addition, at 5 DAT, the combination treatment of *Bb* WG-21 + spinosad caused 91.66% mortality of females, while the single application of each fungal isolate *Bb* WG-21 and *Mr* WG-04 alone caused 51.66 and 62.08% mortality, respectively, and spinosad alone killed 38.75% (Figure 6, Table S1). At 7 DAT, 100% mortality of females was recorded on leaf discs exposed to the *Bb* WG-21 + spinosad combination treatment, whereas treatments with females exposed to the fungal isolates or spinosad treatment alone did not surpass 77.08% (Figure 7, Table S1).

### 3.2. Tetranychus urticae Populations on Tomato Plants in the Greenhouse

**Figure 8:**
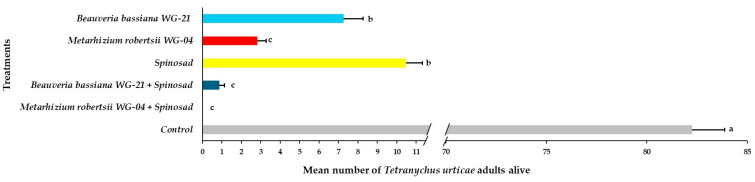
Mean number ± Standard Error (SE) of *Tetranychus urticae* adults alive on the abaxial (bottom) side of tomato leaves after treatment with *Beauveria bassiana* WG-21, *Metarhizium robertsii* WG-04, spinosad, control (water + Tween 80), and in combinations (*Beauveria bassiana* WG-21 + Spinosad and *Metarhizium robertsii* WG-04 + Spinosad) at 21 days after treatment. Different letters indicate significant differences.

**Figure 9:**
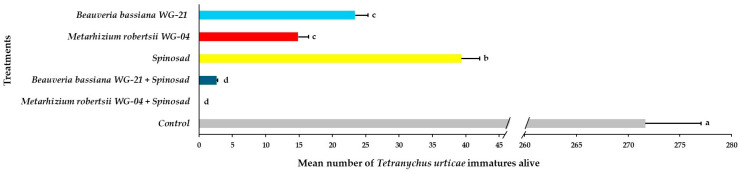
Mean number ± Standard Error (SE) of *Tetranychus urticae* immatures alive on the abaxial (bottom) side of tomato leaves after treatment with *Beauveria bassiana* WG-21, *Metarhizium robertsii* WG-04, spinosad, control (water + Tween 80), and in combinations (*Beauveria bassiana* WG-21 + Spinosad and *Metarhizium robertsii* WG-04 + Spinosad) at 21 days after treatment. Different letters indicate significant differences.

**Figure 10:**
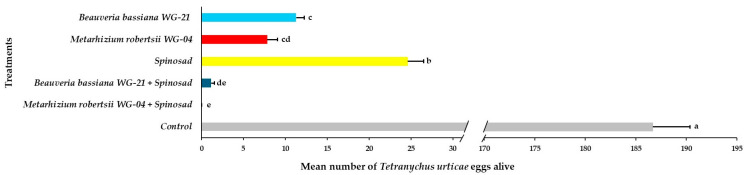
Mean number ± Standard Error (SE) of *Tetranychus urticae* eggs alive on the abaxial (bottom) side of tomato leaves after treatment with *Beauveria bassiana* WG-21, *Metarhizium robertsii* WG-04, spinosad, control (water + Tween 80), and in combinations (*Beauveria bassiana* WG-21 + Spinosad and *Metarhizium robertsii* WG-04 + Spinosad) at 21 days after treatment. Different letters indicate significant differences.

Based on the ANOVA, the main effects and interactions of parameters regarding the presence of *T. urticae* adults, immatures, and eggs on tomato plants following acaricidal treatments in greenhouse trials were significant (Table 2). The number of adults alive on the abaxial side of leaves varied significantly among treated plants compared to control plants 21 DAT (Figure 8, Table S2). No adults were observed on leaves after the application of the *Mr* WG-04 + spinosad combination treatment. A significantly lower number of adults was observed in the combination of *Bb* WG-21 + spinosad (0.87 adults per leaf) and *Mr* WG-04 alone (2.89 adults per leaf) treatments compared to the *Bb* WG-21 (7.26 adults per leaf) and spinosad treatments alone (10.47 adults per leaf). The highest number of adults was found on control plants, with 82.25 adults per leaf compared to the other treatments. Regarding immatures on the bottom (abaxial) surface of the leaves, significantly lower numbers were found in the combination treatments of *Mr* WG-04 + spinosad (0.00 immatures per leaf) and *Bb* WG-21 + spinosad (2.63 immatures per leaf) compared to *Mr* WG-04 (14.87 immatures per leaf), *Bb* WG-21 (23.42 immatures per leaf) and spinosad (39.34 immatures per leaf) applied alone (Figure 9, Table S2). In contrast, 271.67 immatures per leaf were recorded in the control plants, which differed significantly from the treated plants. Concerning the presence of eggs counted on the bottom surface of leaves, control plants had a significantly higher number (186.70 eggs per leaf) compared to spinosad (24.62 eggs per leaf), *Bb* WG-21 (11.29 eggs per leaf), *Mr* WG-04 (7.85 eggs per leaf) alone, and the combination treatments of *Mr* WG-04 + spinosad (0.00 egg per leaf) or *Bb* WG-12 + spinosad (1.15 eggs per leaf) (Figure 10, Table S2).

**Figure 11:**
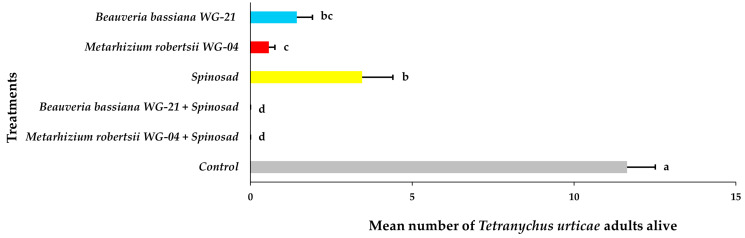
Mean number ± Standard Error (SE) of *Tetranychus urticae* adults alive on the adaxial (top) side of tomato leaves after treatment with *Beauveria bassiana* WG-21, *Metarhizium robertsii* WG-04, spinosad, control (water + Tween 80), and in combinations (*Beauveria bassiana* WG-21 + Spinosad and *Metarhizium robertsii* WG-04 + Spinosad) at 21 days after treatment. Different letters indicate significant differences.

**Figure 12:**
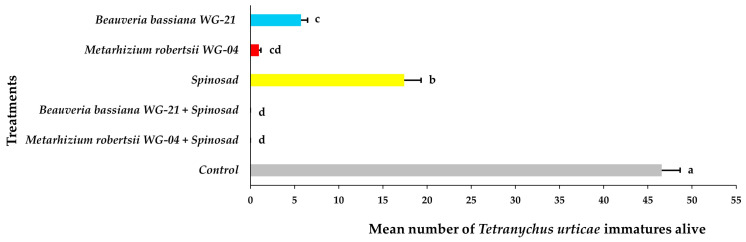
Mean number ± Standard Error (SE) of *Tetranychus urticae* immatures alive on the adaxial (top) side of tomato leaves after treatment with *Beauveria bassiana* WG-21, *Metarhizium robertsii* WG-04, spinosad, control (water + Tween 80), and in combinations (*Beauveria bassiana* WG-21 + Spinosad and *Metarhizium robertsii* WG-04 + Spinosad) at 21 days after treatment. Different letters indicate significant differences.

**Figure 13:**
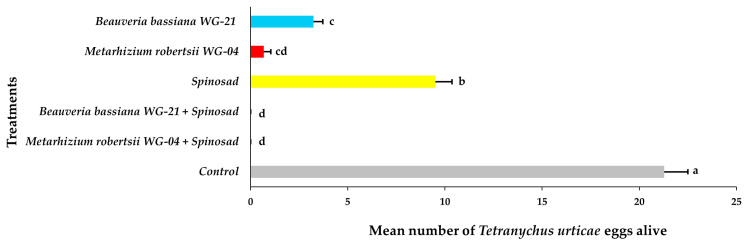
Mean number ± Standard Error (SE) of *Tetranychus urticae* eggs alive on the adaxial (top) side of tomato leaves after treatment with *Beauveria bassiana* WG-21, *Metarhizium robertsii* WG-04, spinosad, control (water + Tween 80), and in combinations (*Beauveria bassiana* WG-21 + Spinosad and *Metarhizium robertsii* WG-04 + Spinosad) at 21 days after treatment. Different letters indicate significant differences.

Similar findings were noted regarding the establishment of *T. urticae* on the top (adaxial) surface of the leaves 21 DAT (Figure 11, Table S2). No adults were recorded for either EPF + spinosad combination treatments. Fewer adults were found on plants treated with *Mr* WG-04 (0.57 adults per leaf) and *Bb* WG-21 (1.43 adults per leaf) than on those treated with spinosad (3.45 adults per leaf) alone compared to the control group, which had the highest number of adults recorded (11.64 adults per leaf). The number of adults counted at 21 DAT in all the treated plants was significantly lower compared to that in the control. No immatures were observed on the adaxial surface of the leaves in those plants treated with *Mr* WG-04 or *Bb* WG-21 combined with spinosad (Figure 12, Table S2). In the individual treatments, the number of immatures was significantly higher in the spinosad treatment (17.42 immatures per leaf) compared to either *Bb* WG-21 (5.74 immatures per leaf) or *Mr* WG-04 (0.96 immatures per leaf). In contrast, significantly more immatures were counted in control plants compared to the treated plants. Similarly, no eggs at 21 DAT were found after being exposed to the combination treatment of *Mr* WG-04 or *Bb* WG-21 plus spinosad. The application of each EPF treatment alone (*Mr* WG-04 or *Bb* WG-21) significantly decreased the number of eggs (0.68 and 3.24 eggs per leaf, respectively) on the adaxial surface when compared to spinosad (9.51 eggs per leaf) and control plants (21.28 eggs per leaf) (Figure 13, Table S2). The number of eggs counted in all treated plants was significantly lower compared to that in the control.

## 4. Discussion

*Beauveria bassiana* and *M. robertsii* isolates have demonstrated considerable potential in the management of *T. urticae* and in providing an effective biological control option as an alternative to the broad-spectrum synthetic acaricides [25,26,27,86,87,88]. In the third day of mortality estimation, *B. bassiana* WG-21 killed 34.58% of *T. urticae* females. This was expected since *B. bassiana* normally takes approximately three days to show acaricidal effects. Wu et al. [89] found that germinated conidia successfully penetrated the cuticle of *T. urticae* adults within 2.5 days. For instance, Wakil et al. [25] reported 100.00% mortality of *T. urticae* female adults after exposure to *M. robertsii* WG-02 and *B. bassiana* WG-12 isolates on leaf discs at 10 DAT, whereas the *B. bassiana* WG-19 isolate achieved 94.58% mortality of exposed females over the same duration. Also, in the current work, both fungal isolates demonstrated nymphal and adult mortality rates exceeding 74.00% and 63.00% at 3 DAT and 7 DAT, respectively, significantly outperforming spinosad on leaf discs. In addition, the *M. robertsii* isolate WG-04 consistently caused significantly higher mortality in both nymphs and female adults of *T. urticae* compared to the *B. bassiana* isolate WG-21 throughout the experimental period under laboratory conditions. Similar findings were reported by Wakil et al. [26], with the WG-7 isolate of *M. robertsii* proving to be more virulent against *T. urticae* nymphs and adults (females) than the WG-12 isolate of *B. bassiana* 7 days after application on leaf discs. Recently, Elhakim et al. [27] reported that *M. robertsii* killed more female adults of *T. urticae* (85.00%) compared to *B. bassiana* (70.00%) 7 days after spraying. The authors attributed the difference in the virulence of both EPF isolates against *T. urticae* to the greater protease activity observed in *M. robertsii* compared to *B. bassiana* [27,90] since this activity constitutes one of the primary mechanisms through which EPF exert their pathogenic effects [27,90,91]. Moreover, protease activity is essential in the host penetration process [27,91,92,93,94]. Interestingly, in our study, nymphs were more sensitive to EPF than adult females. Similar results have been reported by Wakil et al. [26]. However, previous studies have reported contrary results, with adults having a higher susceptibility to various fungal species, including *M. robertsii* and *B. bassiana*, than nymphs [87,95,96]. This high tolerance is explained due to the ecdysis of nymphs, where the spores of EPF are discarded with the cuticle. For instance, Ranout et al. [96] found mycelial development in adult cadavers vs. no mycelial development in nymphal cadavers, indicating that ecdysis took place before fungal sporulation [95,96,97]. Therefore, it becomes evident that different EPF species and isolates exhibit variable virulence in adults and nymphs of *T. urticae*.

Previous studies have explored the combined use of EPF with insecticides as a valuable pest control strategy, highlighting their potential to reduce the risk of insecticide resistance [55,98,99]. For instance, Wakil et al. [26] found that the paired treatments of EPF *B. bassiana* WG-12 and *M. robertsii* WG-7 plus abamectin killed all exposed nymphs and adults (females) of *T. urticae* 3 and 7 d post application. In addition, Wakil et al. [26] found that individual treatments of EPF or abamectin caused significantly lower mortality (i.e., <75 and <65% for immatures and adults, respectively) compared to their respective combination treatments (i.e., 100% for nymphs and adults). Similar findings were obtained in the current study using leaf discs, where the combinations of EPF with spinosad caused 100% nymphal and adult female mortality in the same time interval (3 DAT and 7 DAT, respectively). In addition, the treatments of *B. bassiana* WG-21, *M. robertsii* WG-04, and spinosad alone did not exceed 86.50 and 77.50% mortality for nymphs and adults, respectively. Therefore, it is evident that combination treatments of EPF with different active ingredients can have an additive effect upon EPF efficacy and achieve higher mortality rates compared to the application of each control agent alone against *T. urticae* under laboratory conditions [26]. The slow action of EPF on *T. urticae*, when used alone, may be due to the time needed for fungi to attach, penetrate, germinate, and proliferate in a host haemocoel [27,100]. This type of activity is a barrier to using EPF alone because they need considerable time to suppress the mite population, while the infested crop is being damaged [101]. The environmental conditions also slow down EPF activity. For example, temperatures <16 °C delay the germination and growth of EPF, affecting their long-term virulence [29,102]. To overcome these drawbacks, EPF applications should be repeated and used to manage low densities of pests; however, the overall cost of management will be increased [29]. This is also evident from our results where population establishment of *T. urticae* was not avoided on EPF-treated tomato plants, providing the highest immature population in comparison to the combined treatments.

Similarly, spinosad is a comparatively slow-acting toxicant in comparison to other classes of insecticides (e.g., organophosphates or pyrethroids). Intoxication symptoms of spinosad appear very fast and peak cumulative percent mortality increases gradually [103,104]. This progressive mortality is affected by the slow penetration of the cuticle by spinosad [104]. Sparks et al. [103] reported that spynosin A penetrated into *Heliothis virescens* (F.) (Lepidoptera: Noctuidae) larvae slowly, but when it entered the insect, spynosin A was not easily metabolized by the host either. Sparks et al. [103] noted that this limited metabolism in larvae of *H. virescens* contributed to the observed increased activity against this pest, thereby compensating slower penetration rate of spynosin A. Presumably, the protease activity of EPF [90] accelerated the penetration of spinosad into mites, providing increased rates of mortality faster than the single applications of EPF or spinosad alone. This combined activity may explain the highest and most rapid efficacy of the paired treatment of *M. robertsii* WG-04 + spinosad against *T. urticae*; however, this hypothesis needs to be further investigated. There is no precise report available describing the mechanisms of rapid mortality in combinations of EPF and spinosad. We hypothesized that one agent might stress the pest while enhancing its vulnerability to infection or susceptibility to insecticides by giving an additive effect [34,105,106]. The possible mechanism of EPF–spinosad interaction is that the pesticide weakens the immune system of the target organism [107,108,109,110]. In addition, Rivero-Borja et al. [110] reported that when spinosad application preceded the application of EPF (*M. robertsii* isolate ETL, or *B. bassiana* isolate Bb88), fungal performance improved in comparison to the case where EPF preceded the application of the pesticide. In our study, spinosad was applied first, followed by the application of EPF.

Concerning the establishment of *T. urticae* on tomato plants, the mites predominantly prefer to inhabit and feed on the abaxial surface of leaves [26,111,112,113]. The findings from this study revealed a greater number of *T. urticae* on the abaxial surface of the tomato leaves compared to the adaxial side, where the application of each treatment significantly suppressed the presence of mites compared to the control. However, most importantly, the combination treatment of *M. robertsii* WG-04 + spinosad completely prevented the establishment of *T. urticae* on either side of the leaves. In plants treated with *B. bassiana* WG-21 + spinosad, only a few mites were found in the abaxial surface of leaves (i.e., <2.70 individuals/leaf). However, in contrast to the combination treatments, the least effective treatment was spinosad on either side of the leaf surface. In corroboration, Wakil et al. [26] observed similar results, where the combination treatments of *M. robertsii* WG-7 or *B. bassiana* WG-12 plus abamectin significantly reduced *T. urticae* presence on both leaf surfaces, whereas abamectin alone showed lower efficacy than either of the combination treatments. Therefore, plant protection and potentially increased yield can be achieved after the combined application of EPF with a compatible acaricide, especially the combination treatments of *M. robertsii* + spinosad (current study) and abamectin combinations [26], which can cause a significant reduction of *T. urticae* populations.

The outcome of this study indicates that *B. bassiana* WG-21 + spinosad or *M. robertsii* WG-04 + spinosad combination treatments are effective tools against various life stages of *T. urticae*. The combination treatments were more efficacious compared to the application of each component alone, which could be due to their rapid activity, increased mortality rates, and resultant low pest populations. It is widely recognized that when two agents with different modes of action are combined, their activity may be independent (additive effect) or one agent may induce sufficient stress on the pest, boosting the effectiveness of the second agent (synergistic effect) [99,107,114]. Furthermore, since different isolates of *M. robertsii* and *B. bassiana* have shown varying mortality rates against *T. urticae* [25,26], the selection of appropriate fungal isolates is essential for the efficacy of EPF against pests [10,27], especially when used in combination with a compatible pesticide. Thus, based on the results of this study, the use of new entomopathogenic fungal isolates from different geographical areas in combination with spinosad may be a promising alternative strategy for managing this mite pest successfully.

## Figures and Tables

**Table 1:** ANOVA parameters for mortality rates of *Tetranychus urticae* exposed to tomato leaf discs treated with *Metarhizium robertsii*, *Beauveria bassiana*, or spinosad, alone and in combination (Total df = 539).

Source	df	*F*	*p*
Interval	2	687.77	<0.01
Life stage	1	266.74	<0.01
Treatment	4	1550.48	<0.01
Interval × life stage	2	1.81	0.16
Interval × treatment	8	15.60	<0.01
Life stage × treatment	4	14.13	<0.01
Interval × stage × treatment	8	3.33	<0.01

**Table 2:** ANOVA parameters for the number of individuals (adults, immatures, eggs) of *Tetranychus urticae* alive on tomato leaves after application with EPF *Beauveria bassiana* (*Bb* WG-21), *Metarhizium robertsii* (*Mr* WG-04) or spinosad alone, and in combination. (Total df = 431).

Source	df	*F*	*p*
Leaf side (abaxial or adaxial)	1	3978.95	<0.01
Life stage	2	852.20	<0.01
Treatment	5	4337.09	<0.01
Leaf side × life stage	2	356.22	<0.01
Leaf side × treatment	5	2439.46	<0.01
Life stage × treatment	10	421.93	<0.01
Leaf side × life stage × treatment	10	207.11	<0.01

## Data Availability

The data are contained within this article.

## Supplementary Materials

The following supporting information can be downloaded at: https://www.mdpi.com/article/10.3390/jof11020138/s1, Table S1: Mean mortality % ± Standard Error (SE) of nymphs and adult females of *Tetranychus urticae* exposed to tomato leaf discs after the treatment with the entomopathogenic fungi *Beauveria bassiana* (*Bb* WG-21), *Metarhizium robertsii* (*Mr* WG-04) or spinosad, and in combinations (*Bb* WG-21 + Spinosad and *Mr* WG-04 + Spinosad) at different days after treatment. Within each row, means with the same uppercase letter are not significantly different (df = 2.35, Tukey HSD test at *p* = 0.05). Within each column, means followed by the same lowercase letter are not significantly different (df = 4.59, Tukey HSD test at *p* = 0.05); Table S2: Mean number ± Standard Error (SE) of individuals of *Tetranychus urticae* alive at different life stages (adults, immatures, eggs) on the abaxial and adaxial side of tomato leaves after treatment with *Beauveria bassiana* (*Bb* WG-21), *Metarhizium robertsii* (*Mr* WG-04), spinosad, control (water + Tween 80), and the respective paired combinations of the entomopathogenic fungi with spinosad (*Bb* WG-21 + Spinosad and *Mr* WG-04 + Spinosad) per leaf side at 21 days after treatment. Within each row means with the same uppercase letter(s) are not significantly different (df = 2.35, Tukey HSD test at *p* = 0.05). Within each column, means followed by the same lowercase letter are not significantly different (df = 5.71, Tukey HSD test at *p* = 0.05). Where dashes exist, no statistics were applied.
