# Supplementary material for: The Inevitable Fate of Tetranychus urticae on Tomato Plants Treated with Entomopathogenic Fungi and Spinosad
Source: J Fungi (Basel). 2025 Feb 12;11(2):138. doi: 10.3390/jof11020138 (PMC11856040; doi:10.3390/jof11020138)
Supplement: Supplementary file 1 [file jof-11-00138-s001.zip › jof-3467457-supplementary.pdf]

Table S1. Mean mortality % ± Standard Error (SE) of nymphs and adult females of *Tetranychus urticae* exposed to tomato leaf discs after the treatment with the entomopathogenic fungi *Beauveria bassiana* (*Bb* WG-21), *Metarhizium robertsii* (*Mr* WG-04) or spinosad, and in combinations (*Bb* WG-21 + Spinosad and *Mr* WG-04 + Spinosad) at different days after treatment. Within each row, means with the same uppercase letter are not significantly different (df = 2, 35, Tukey HSD test at p = 0.05). Within each column, means followed by the same lowercase letter are not significantly different (df =4, 59, Tukey HSD test at p = 0.05).

Life Stage	Treatment	Day(s) after treatment			*F*	*p*
		1	2	3		
Nymphs	*Bb* WG-21	47.91±1.29Cd	65.41±1.78Bc	74.16±1.82Ac	65.1	< 0.01
	Mr WG-04	56.25±1.39Cc	73.33±1.54Bb	86.25±2.54Ab	62.7	< 0.01
	Spinosad	32.50±1.89Ce	49.16±1.20Bd	62.91±1.68Ad	88.4	< 0.01
	Bb WG-21 + Spinosad	83.75±1.39Bb	100.00±0.00Aa	100.00±0.00Aa	136.1	< 0.01
	Mr WG-04 + Spinosad	92.08±1.43Ba	100.00±0.00Aa	100.00±0.00Aa	30.3	< 0.01
	F	276.0	351.2	104.6		
	p	< 0.01	< 0.01	< 0.01		
		3	5	7		
Females (adults)	*Bb* WG-21	34.58±1.43Cd	51.66±1.54Bd	63.75±2.31Ac	65.6	< 0.01
	Mr WG-04	45.83±1.20Cc	62.08±1.89Bc	77.08±1.29Ab	109.3	< 0.01
	Spinosad	23.75±1.64Ce	38.75±1.39Be	46.25±1.39Ad	59.8	< 0.01
	Bb WG-21 + Spinosad	74.58±1.43Cb	91.66±1.66Bb	100.00±0.00Aa	104.5	< 0.01
	Mr WG-04 + Spinosad	85.41±1.68Ba	100.00±0.00Aa	100.00±0.00Aa	75.3	< 0.01
	F	311.9	320.1	303.2		
	p	< 0.01	< 0.01	< 0.01		

Table S2. Mean number ± Standard Error (SE) of individuals of *Tetranychus urticae* alive at different life stages (adults, immatures, eggs) on the abaxial and adaxial side of tomato leaves after treatment with *Beauveria bassiana* (*Bb* WG-21), *Metarhizium robertsii* (*Mr* WG-04), spinosad, control (water + Tween 80), and the respective paired combinations of the entomopathogenic fungi with spinosad (*Bb* WG-21 + Spinosad and *Mr* WG-04 + Spinosad) per leaf side at 21 days after treatment. Within each row means with the same uppercase letter(s) are not significantly different (df = 2, 35, Tukey HSD test at $p = 0.05$). Within each column, means followed by the same lowercase letter are not significantly different (df =5, 71, Tukey HSD test at $p = 0.05$). Where dashes exist, no statistics were applied.

Leaf side	Treatment	Number of *Tetranychus urticae* alive			*F*	*P*
		Adults	Immatures	Eggs		
Abaxial (Bottom)	*Bb* WG-21	7.26±1.01Bb	23.42± 1.95Ac	11.29±0.96Bc	36.7	< 0.01
	Mr WG-04	2.89±0.46Cc	14.87±1.55Ac	7.85±1.22Bcd	26.3	< 0.01
	Spinosad	10.47±0.84Cb	39.34±2.72Ab	24.62±1.87Bb	53.7	< 0.01
	Bb WG-21 + Spinosad	0.87±0.26Bc	2.63±0.18Ad	1.15±0.40Bde	9.86	< 0.01
	Mr WG-04 + Spinosad	0.00±0.00c	0.00±0.00d	0.00±0.00e	-	-
	Control	82.25±1.62Ca	271.67±5.37Aa	186.70±3.68Ba	599.1	< 0.01
	F	1320.9	1564.2	1630.8		
	P	< 0.01	< 0.01	< 0.01		
Adaxial (Top)	*Bb* WG-21	1.43±0.49Bbc	5.74±0.74Ac	3.24±0.48Bc	13.5	< 0.01
	Mr WG-04	0.57±0.19Ac	0.96±0.24Acd	0.68±0.36Acd	0.53	0.59
	Spinosad	3.45±0.95Cb	17.42±1.92Ab	9.51±0.86Bb	27.5	< 0.01
	Bb WG-21 + Spinosad	0.00±0.00c	0.00±0.00d	0.00±0.00d	-	-
	Mr WG-04 + Spinosad	0.00±0.00c	0.00±0.00d	0.00±0.00d	-	-
	Control	11.64±0.87Ca	46.57±2.08Aa	21.28±1.22Ba	147.3	< 0.01
	F	61.9	231.7	162.9		
	P	< 0.01	< 0.01	< 0.01		
